# Time to death predictors of HIV/AIDS infected patients on antiretroviral therapy in Ethiopia

**DOI:** 10.1186/s13104-018-3863-y

**Published:** 2018-10-25

**Authors:** Melaku Tadege

**Affiliations:** Department of Statistics, Injibara University, Injibara, Amhara Ethiopia

**Keywords:** HIV, ART, TB, Survival

## Abstract

**Objective:**

The purpose of this study was to identify the major risk factors, which contributed to shortened survival time to death of HIV patients on antiretroviral therapy. Six-hundred HIV patients were included from two hospitals and six health centers record from January 2003 to December 2017. Kaplan–Meier and Cox proportional hazard model were implemented.

**Results:**

From the Kaplan–Meier, log-rank test result indicated that there was a significant difference between tuberculosis comorbidity (P = .000), occupation (P = .027), and WHO clinical stage (P = .012) on the survival experience of patients at 5% statistical significance level. From the Cox regression result, the risk of death for patients who lived with tuberculosis was about 2.872-fold times higher than those patients who were negative. Most of the HIV/AIDS patients on antiretroviral therapy were died in a short period due to tuberculosis comorbidity, began with lower amount of CD4, being underweight, merchant, and being on WHO clinical stage IV.

**Electronic supplementary material:**

The online version of this article (10.1186/s13104-018-3863-y) contains supplementary material, which is available to authorized users.

## Introduction

Human immunodeficiency virus is a cause of reducing a person’s ability to fight infection by reducing CD4 cell which is responsible for the body’s immune response to infectious agents [1]. Human immunodeficiency virus is a public health problem globally. In 2013, the United Nations program on HIV/AIDS documented that there were about 35.3 million individuals living with HIV/AIDS [2]. Nearly two million People were died because of AIDS-associated causes worldwide, 70% occurred in sub-Saharan Africa [3]. HIV incidence accounted for around 70% of all new HIV infections [4].

Sub-Saharan Africa is one of the most affected parts of the world with about 22.9 million people living with HIV AIDS and 1.2 million deaths from AIDS among children and adults in 2010 [5]. In Ethiopia, approximate to one million people are living with HIV which become the leading cause of mortality among 15–49 years of age, that accounts for about 43% of all population death in 2008 [6]. The previous study in 2012 estimated that Ethiopia is one of the most affected parts of sub-Saharan African countries by HIV and there were 41,444 deaths because of HIV/AIDS from 2011 to 2016 [7]. The previous studies discussed that discontinue ART will contribute to the death incidence of patients [8].

The approximate total 114,690 Ethiopian’s died of AIDS-related conditions and this increased the number of children who lost their parents due to HIV/AIDS. As a result, this figure will also increase infant mortality rate and affect the population size in Ethiopia. In addition to mortality, the HIV/AIDS disease in Ethiopia has decreased the country’s developmental growth. HIV/AIDS is influencing every sector. This evaluation will be below the reality estimation because it is difficult to get the exact figure of death and incidence rate in the country, Ethiopia [9].

There were statements which explained that there was HIV improvement in Ethiopia even though there are a lot of people living with HIV/AIDS, death prevalence, and the economic effect in the country level are increasing. Some hospital based survey showed that several patients live with HIV and its mortality was high in Illubabor and Buno Bedele Zones. To this effect, the objective of this study was to determine predictors which contributed to shortened survival time to death of HIV/AIDS patients.

## Main text

### Methods

#### Study area and period

Illubabor and Buno Bedele Zones are parts of Oromia Regional State placed in the Southwest part of Ethiopia which is 600 km far from Addis Ababa and a well known place for coffee production, evergreen forest and a variety of tourist attractions such as rivers. Based on the 2007 census conducted by the central statistical agency, the two Zones have a total population of 1,271,609. The study was run from January 2003 to December 2017. This study was covered six health providing centers and two senior hospitals from Illubabor and Buno Bedele Zones.

#### Design of study

The retrospective cohort study design was applied on patients with HIV/AIDS on ART services in six HIV/AIDS health centers and two senior hospitals of Illubabor and Buno Bedele Zones from 2003 to 2017.

#### Study population

All adult HIV/AIDS patients on ART service in Ethiopia was the target population for this study and the subject population was on ART service from January 2003 to December 2017 in two senior hospitals and six health providing centers of Illubabor and Buno Bedele Zones of Oromia Region. Patients above 16 years old were covered in the study. Patients with incomplete variable of interest as well as dropout from ART were excluded.

#### Sampling technique and procedure

**Fig. 1:**
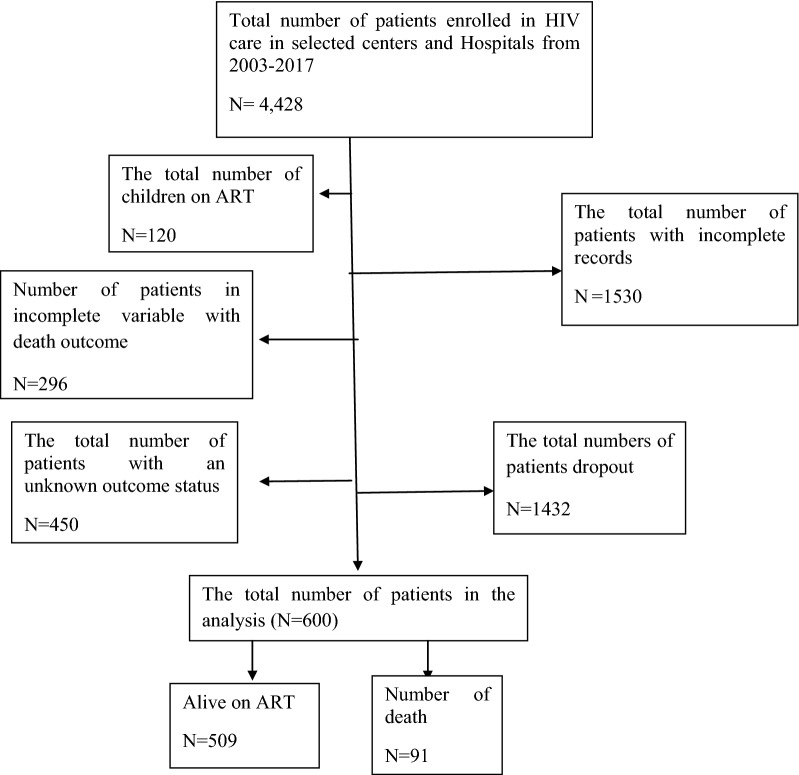
Sampling procedure. Sample selection extraction (sample to be included in the study)

The study was applied purposive sampling technique to include the two Zones from the 21 Zones of Oromia Region. From all HIV/AIDS ART providing clinics in Illubabor and Buno Bedele Zones, eight health providers (two senior hospitals and six ART providing centers) were selected for the study. The two hospitals called Karl hospital from Illubabor and Bedele hospital from Buno Bedele Zones were included in the study. Those six health centers were selected through simple random sampling technique. Thus, one senior hospital and three health center were selected from each Zone to be included in the study. Sample selection process was explained in Fig. 1. Six hundred patients on ART from the selected clinics were included as the study population. Here each patient has a record with a distinctive identification number. The data was collected by health professionals and closely followed by the researcher throughout the entire data collection. Two data clerks did the data entry process into the statistical package of social science (SPSS) software.

#### Study variables

The dependent variable was time to death and divided as death (event) and censored outcomes which measured in months. Censored refers to alive. The purposes of this investigation, particularly the times for the patients who were died treated as event times and other outcomes assumed as right-censored.

The predictor variables included in the study were age, sex (male, female), marital status (married, divorced, separated, widow and never married), religion (Orthodox, Protestant, and Muslim), occupation (wife, daily labour, farmer, government worker, merchant), WHO clinical stage (I, II, III, Iv), baseline regimen type (AZT-3TC-NVP, D4t-3TC-NVP, D4t-3TC-EFV, AZT-3TC-EFV), tuberculosis co-infection (positive, negative) and educational level (illiterate, primary, secondary and above secondary), CD4 level, and body weight.

#### Data analysis

Statistical analyses were performed using STATA version 12, statistical package of social science (SPSS) version 20 and SAS 9.2 statistical softwares (see Additional file 1). Kaplan–Meier estimators were used to display the survival experience of patient’s overtime for categorical predictor variables. The log-rank test was applied to test whether there is a significant difference between predictor variables towards the survival ability of patients. Cox proportional hazard model (h(t) = h_0_(t) * exp (b_1_x_1_ + b_2_x_2_ + ⋯ +b_p_x_p_) were used to identify and to check the impact of each independent variables on the time to death event. Hazard function h(t) is determined by a set of p covariates (x_1_, x_2_, …, x_p_), whose impact is measured by the size of the respective coefficients (b_1_, b_2_,…, b_p_). The term h_0_(t) is called the baseline hazard for a time to death. The proportionality assumptions of Cox model were assured on all each predictor and on the general test of proportionality. None of the predictors was failed Cox proportional hazard model required assumption (see Additional file 2). The result of Wald, Likelihood, and Score test had P (< .05) and the R^2^ adjusted residual value (.019) explained the data and the model were in a good fit.

### Results

#### Demographic characteristics of patients

From the total of 600 HIV patients (59.8%) and (45.8%) of them were female and had a primary school education level respectively. On the subject of WHO clinical stage; (57.2%), (20.0%), (17%) and (5.8%) of patients were stage III, II, I and IV, respectively. Regarding tuberculosis comorbidity, (19.5%) of patients were TB positive (see Additional file 3).

#### Chi square test of association

There were 600 patients in the cohort study out of which 91 (18%) died under antiretroviral therapy. Proportional to death for a patient who had baseline TB comorbidity were greater than those patients who were negative. Chi square test shows that death status is associated with religion, WHO clinical stage, and TB comorbidity (P < .05) (see Additional file 4).

#### Comparison of the survival ability of patients

From the Kaplan–Meier survivor estimate, merchants had shortened meantime as compared to the housewife, daily labour, the government employed and farmers. Kaplan–Meier survival estimates for the TB comorbidity group explained that TB positive had shortened mean death time as compared with TB negative. More generally from the log-rank test, there was a significant difference between TB status groups (P = .000), occupation (P = .027), religion (P = .038), and WHO clinical stage (P = .012) on the death time of HIV/AIDS patients on ART (Table 1).

**Table 1:** Survival experience comparison

Variables	Mean time	95% CI for mean time		Log-rank P value
		Lower	Upper	
Sex				
Female	119.112	114.630	123.593	.157
Male	126.618	119.340	133.896	
Marital status				
Married	114.237	108.919	119.555	.675
Divorced	121.684	110.192	133.177	
Separated	119.538	111.653	127.422	
Widow	128.734	116.752	140.716	
Never married	123.394	110.810	135.978	
Education				
Illiterate	110.514	99.941	121.088	.502
Primary school	123.134	117.917	128.351	
Secondary school	114.937	107.545	122.329	
Above secondary	129.451	118.130	140.772	
Religion				
Orthodox	125.102	118.708	131.497	.038
Protestant	130.021	123.672	136.371	
Muslim	118.465	110.794	126.137	
Occupation				
Wife	121.142	115.294	126.990	.027
Daily labour	113.575	104.588	122.561	
Farmer	119.675	111.432	127.919	
Government worker	126.332	116.715	135.948	
Merchant	100.666	85.705	115.627	
WHO clinical stage				
Stage I	121.904	115.402	128.405	.012
Stage II	121.587	114.878	128.296	
Stage III	118.618	112.951	124.284	
Stage IV	123.851	105.592	142.109	
Original regimen				
D4t-3TC-NVP	118.788	113.000	124.576	.726
D4t-3TC-EFV	114.276	100.444	128.107	
AZT-3TC-NVP	133.974	127.365	140.582	
AZT-3TC-EFV	111.694	100.202	123.186	
Tuberculosis comorbidity				
Positive	97.322	85.538	109.105	.000
Negative	136.786	132.558	141.015	

#### Single covariate analysis

The relationship between each covariate and death time of HIV patients on antiretroviral therapy are presented in Table 2. As seen from this table, time to death is related to baseline CD4 level, occupation, TB status, baseline weight and WHO clinical stage at 5% of the significant level.

**Table 2:** Single covariate and multiple covariates analysis

Variables	Single covariates result		Multiple covariate results	
	Crudes HR (95% CI for its HR)	P	Adjusted HR (95% CI for its HR)	P
Occupation				
Wife	.397 (.206–.762)	.038*	.381 (.193–.754)	.078
Daily labour	.609 (.304–1.219)		.654 (.324–1.319)	
Farmer	.346 (.152–.792)		.523 (.225–1.216)	
Government worker	.617 (.328–1.159)		.681 (.359–1.290)	
Merchant				
CD4	.994 (.991–.997)	.000*	.995 (.993–.998)	.000*
Weight	.947 (.921–.973)	.000*	.952 (.925–.979)	.001*
WHO clinical stage				
Stage I	.321 (.116–.887)	.019*	.335 (.119–.941)	.023*
Stage II	.448 (.183–1.099)		.336 (.134–.839)	
Stage III	.859 (.412–1.793)		.690 (.324–1.472)	
Stage IV (ref)				
TB				
Positive	3.439 (2.269–5.213)	.00*	2.872 (1.870–4.409)	.000*
Negative (ref)				

*AHR* adjusted hazard ratio, *TB* tuberculosis

*Statistical significance, ref = reference category and CI means confidence interval of the hazard ratio estimate

#### Multi covariate analysis

The probability of death for patients with world health organization stage one .335 times lower than those patients who were world health organization stage IV [adjusted HR = .335, CI (.119–.941)]. The risk of death for patients who lived in TB was about 2.872-fold times higher than those patients who were negative [AHR = 2.872, CI (1.870–4.409)]. When the CD4 count increased by 1 unit, the risk of death was decreased by .995 times [AHR = .995, CI (.993–.998)]. The single KG increment of weight had a power to decrease the risk of death into 4.8% [AHR = .952, CI (.925–.979)] (see Table 2 below).

### Discussion

In this survival retrospective cohort study, there were 91 deaths from 600 patients, yielding death prevalence density are around 16 out of 100. The independent predictors of mortality were WHO clinical stage, low weight, low CD4 count and TB co-infection. The estimated survival probability live of the cohort in 14 years were 84%. This shows almost equal survival experience as compared to other studies in the Africa continent. In a Malawian cohort study, the average of the probability of being alive on antiretroviral therapy was 84% [10]. The result from this study shows patients with higher CD4 level have a smaller risk of mortality [AHR: .995 (.993–.998)], which is directly related with the study [11–14]. A 1 kg weight increase cause to reduce mortality [AHR: .952 (.925–.979)] into 4.8%. Another study in Malawi showed that individuals who were underweight had 6 times higher risk of dying in a short period of time [15]. Body mass index may be affected by late WHO clinical stage AIDS conditions [16, 17]. Patients with TB comorbidity was highly associated with an increased risk of mortality [AHR: 2.872 (1.870–4.409)]. Another investigation in Uganda showed that the overall risk for death related with TB was 1.81 (95% CI 1.24–2.65) [18]. A study in Ethiopia also explained a similar relationship [19]. Death in patients living with HIV in developing countries was linked to coexistent TB infection [20]. The study will serve for policymakers to formulate a better management of HIV/AIDS patients. Additionally, the study will be used as a baseline for further researchers or investigators.

### Conclusion

In conclusion, of all the covariates, TB comorbidity, begin with a low amount of CD4, underweight, being merchant, and level of WHO clinical stage IV were found to be the most influential factor for time to death event.

## Limitation of the study

This study includes only baseline variables. As a result, an investigation with time-varying covariates is recommended. Another drawback of the study was, there are a lot of patients with dropout status from treatment this may have an impact on the study result. Consequently, an investigation with the reason behind dropout from ART is also highly appreciated.

## Additional files

**Additional file 1.** Syntax for Cox proportional hazard model (SAS version 9.2). Cox regression model syntax in SAS.

**Additional file 2.** Test of proportional-hazards assumption (STATA version 12). The global test explained that proportionality assumptions were satisfied.

**Additional file 3.** Socio demographic characteristics of HIV patients in Illubabor and Buno Bedele Zones (SPSS version 20). The descriptive statistics explanation.

**Additional file 4.** Chi square test of association. Test of association between predictor variables and survival status.

## Abbreviations

TB: tuberculosis
HR: hazard ratio
WHO: World Health Organization
HIV: human immunodeficiency virus
AIDS: acquired immune deficiency syndrome
