# Supplementary material for: Time to death predictors of HIV/AIDS infected patients on antiretroviral therapy in Ethiopia
Source: BMC Res Notes. 2018 Oct 25;11:761. doi: 10.1186/s13104-018-3863-y (PMC6202867; doi:10.1186/s13104-018-3863-y)
Supplement: Supplementary file 1 — Additional file 1. Syntax for Cox proportional hazard model (SAS version 9.2). Cox regression model syntax in SAS. [file 13104_2018_3863_MOESM1_ESM.docx]

Syntax for Cox proportional hazard model (SAS version 9.2).

Data y;

Input lists all variables;

Cards;

Past data/enter data

;

Run;

Proc print data=y;

Run;

Proc phreg data=y;

Class lists all categorical variables;

Model time*Status ( event) = list all variable names;

Run;
