# Supplementary material for: Time to death predictors of HIV/AIDS infected patients on antiretroviral therapy in Ethiopia
Source: BMC Res Notes. 2018 Oct 25;11:761. doi: 10.1186/s13104-018-3863-y (PMC6202867; doi:10.1186/s13104-018-3863-y)
Supplement: Supplementary file 2 — Additional file 2. Test of proportional-hazards assumption (STATA version 12). The global test explained that proportionality assumptions were satisfied. [file 13104_2018_3863_MOESM2_ESM.docx]

Test of proportional-hazards assumption (STATA version 12)

----------------------------------------------------------------

Chi2 DF Prob>chi2

---------------------------------------------------------------

Global test 5.34 5 0.3756

----------------------------------------------------------------

Note: Chi2 refers to chi-square statistic value, DF refers to degree of freedom, Prob >chi2 refers to P-value.
