# Supplementary material for: Time to death predictors of HIV/AIDS infected patients on antiretroviral therapy in Ethiopia
Source: BMC Res Notes. 2018 Oct 25;11:761. doi: 10.1186/s13104-018-3863-y (PMC6202867; doi:10.1186/s13104-018-3863-y)
Supplement: Supplementary file 3 — Additional file 3. Socio demographic characteristics of HIV patients in Illubabor and Buno Bedele Zones (SPSS version 20). The descriptive statistics explanation. [file 13104_2018_3863_MOESM3_ESM.docx]

Socio demographic characteristics of HIV patients in Illu Aba Bor and Buno Bedele Zones (SPSS version 20)

| variable | | Frequency | Percent (%) |
| --- | --- | --- | --- |
| Sex | Female | 359 | 59.8 |
|  | Male | 241 | 40.2 |
| Education | No education | 93 | 15.5 |
|  | Primary school | 275 | 45.8 |
|  | Secondary school | 143 | 23.8 |
|  | Tertiary | 89 | 14.8 |
| Occupation | Wife | 194 | 32.3 |
|  | Daily labour | 100 | 16.7 |
|  | Farmer | 90 | 15.0 |
|  | Government worker | 158 | 26.3 |
|  | Merchant | 58 | 9.7 |
| Original regimen type | D4t-3TC-NVP | 278 | 46.3 |
|  | D4t-3TC-EFV | 41 | 6.8 |
|  | AZT-3TC-NVP | 212 | 35.3 |
|  | AZT-3TC-EFV | 69 | 11.5 |
| WHO clinical stage | Stage I | 102 | 17.0 |
|  | Stage II | 120 | 20.0 |
|  | Stage III | 343 | 57.2 |
|  | Stage IV | 35 | 5.8 |
| TB comorbidity | Positive | 117 | 19.5 |
|  | Negative | 483 | 80.5 |
| Marital Status | Married | 309 | 51.5 |
|  | Divorced | 43 | 7.2 |
|  | Separated | 114 | 19.0 |
|  | Widow | 75 | 12.5 |
|  | Never married | 59 | 9.8 |
| Religion | Orthodox | 320 | 53.3 |
|  | Protestant | 166 | 27.7 |
|  | Muslim | 114 | 19.0 |
