# Supplementary material for: Time to death predictors of HIV/AIDS infected patients on antiretroviral therapy in Ethiopia
Source: BMC Res Notes. 2018 Oct 25;11:761. doi: 10.1186/s13104-018-3863-y (PMC6202867; doi:10.1186/s13104-018-3863-y)
Supplement: Supplementary file 4 — Additional file 4. Chi square test of association. Test of association between predictor variables and survival status. [file 13104_2018_3863_MOESM4_ESM.docx]

Chi-square test of association

| Variables | | Death Status | | Chi-Square P-value |
| --- | --- | --- | --- | --- |
|  |  | Death (1) | Censored (2) |  |
| Sex | Female | 49(13.6%) | 310(86.4%) | 0.206 |
|  | Male | 42(17.4%) | 199(82.6%) |  |
| Marital Status | Married | 50(16.2%) | 259(83.8%) | 0.731 |
|  | Divorced | 4(9.3%) | 39(90.7%) |  |
|  | Separated | 15(13.2%) | 99(86.8%) |  |
|  | Widow | 13(17.3%) | 62(82.7%) |  |
|  | Never married | 9(15.3%) | 50(84.7%) |  |
| Educational Level | No education | 18(19.4%) | 75(80.6%) | 0.512 |
|  | Primary school | 36(13.1%) | 239(86.9%) |  |
|  | Secondary school | 23(16.1%) | 120(83.9%) |  |
|  | Tertiary | 14(15.7%) | 75(84.3%) |  |
| Religion | Orthodox | 60(18.8%) | 260(18.8%) | 0.029 |
|  | Protestant | 17(10.2%) | 149(89.8%) |  |
|  | Muslim | 14(12.3%) | 100(87.7%) |  |
| occupation | Wife | 23(11.9%) | 171(88.1%) | 0.052 |
|  | Daily labour | 17(17.0%) | 83(83.0%) |  |
|  | Farmer | 9(10.0%) | 81(90.0%) |  |
|  | Government worker | 27(17.1%) | 131(82.9%) |  |
|  | Merchant | 15(25.9%) | 43(74.1%) |  |
| WHO Clinical stage | Stage I | 7(6.9%) | 95(93.1%) | 0.005 |
|  | Stage II | 12(10.0%) | 108(90.0%) |  |
|  | Stage III | 64(18.7%) | 279(81.3%) |  |
|  | Stage IV | 8(22.9%) | 27(77.1%) |  |
| Regimen | D4t-3TC-NVP | 44(15.8%) | 234(84.2%) | 0.775 |
|  | D4t-3TC-EFV | 7(17.1%) | 34(82.9%) |  |
|  | AZT-3TC-NVP | 28(13.2%) | 184(86.8%) |  |
|  | AZT-3TC-EFV | 12(17.4%) | 57(82.6%) |  |
| TB | Positive | 40(34.2%) | 77(65.8%) | 0.00 |
|  | Negative | 52(10.8%) | 431(89.2%) |  |
